# 

**Published:** 1879-08-20

**Authors:** 


### TABLE OF CONTENTS.

ORIGINAL AND SELECTED ARTICLES.

Continuity Theory in Diphtheria—Test Abrasions, by A. F. Kinne, M.D., 281. Encapsuled Placental Tumor Extracted from the Uterus Five Weeks after Parturition, by H. G. Hobbs, M.D., 284. Empyema or Suppurative Pleuritis, by F. M. Rushing, M.D., 286. DeKalb County Medical Protective Society, 288. Gonorrhea—How to cure It Easily and Quickly, by Dr. H. M. Lawson; Resuscitation of a Young Girl Apparently Dead from Drowning, by E. L. Godfrey, M.D., 290. Sunstroke, 292. Protoxide of Nitrogen for Producing Prolonged Insensibility, 294. Zinc *vs.* Quinine, by W. Gibbon Carter, M.D., 296.

ABSTRACTS AND GLEANINGS.

Codeia as a Sedative, 297. Dextro-Quinine, 298. Cholera Infantum, 299. Prophylaxis in Yellow Fever, 300. Warm Water in Labor, 301. Extraction of Cataract, 303. Syphilis, 304. Oxalate of Cerium in Whooping-Cough, 305. Rhus Poisoning, 306. Phlyctenular Ulcers of the Cornea, 207. Typhoid Fever in Infancy; Short Forceps Superseded, 308. New Remedy in Dyspnœa; Explosive Mixture; Massage of the Tonsils, 309. Discoverer of Anæsthesia; Œsophageal Sound in Hiccough; Peach Kernels for clearing Water; Ergot *vs.* Lactation; Apiol in Amenorrhea and Dysmenorrhea; Facetiæ, 310.

SCIENTIFIC ITEMS.

How Old is the World? How Deeply Does the Earth Quake? 311. Sphymograph; The Tongue and the Sense of Taste; New Scale; Nickel, 312.

PRACTICAL NOTES AND FORMULÆ.

Skin Diseases; Palatable Castor Oil, 313. Misturæ, 314. Hog Cholera; Malarial Fever of the South; Potato and Diphtheria, 315. Dressing Head Wounds; Diarrhœa of Children; Milk Diet in Acute Rheumatism; Ergotin Hypodermics in Epistaxis; To Test for Albumen, 316.

EDITORIAL AND MISCELLANEOUS.

Editorial Notices, 317. Book Notices; Special Notices, 318. Gratuities of Medical Men—An Appeal to the Legislature, 319. Receipted, 320.

